# Effects of Adding Hydroxytyrosol to the Diet of Pigs in the Nursery Phase on Growth Performance, Biochemical Markers, and Fatty Acid Profile

**DOI:** 10.3390/ani15152268

**Published:** 2025-08-01

**Authors:** Rafael Domingos Augusto Rofino, Cassio Antonio Ficagna, Taeline Zamboni, Bruna Klein, Enrico A. Altieri, Kevin E. O’Connor, Reeta Davis, Margaret Walsh, Fernando de Castro Tavernari, Marcel Manente Boiago, Aleksandro Schafer da Silva, Diovani Paiano

**Affiliations:** 1 Postgraduate Program in Animal Science, Santa Catarina State University (UDESC), Chapecó 89815-630, SC, Brazil; rafael.rofino@uzambeze.ac.mz (R.D.A.R.); aleksandro.silva@udesc.br (A.S.d.S.); 2 Department of Animal Engineering, Zambeze University, Mocuba P.O. Box 369, Mozambique; 3 Department of Food and Chemical Engineering, Santa Catarina State University (UDESC), Pinhalzinho 89870-000, SC, Brazil; 4 Nova Mentis Limited, Nova UCD, Belfield Innovation Park, University College Dublin, Belfield, D04 F438 Dublin, Ireland; 5 Empresa Brasileira de Pesquisa Agropecuaria (EMBRAPA-Suínos e Aves), Concórdia 89715-899, SC, Brazil

**Keywords:** antioxidant additive, functional feed additive, lipid composition, piglet nutrition, weaning pigs

## Abstract

This study evaluated the effects of dietary hydroxytyrosol (HT) addition on piglets during the nursery phase across two experiments. In the first, 72 weaned male piglets (~26 days old, 7.3 ± 0.5 kg) were assigned to one of four diets containing 0, 5, 10, or 50 mg HT/kg feed. Growth performance, serum biochemistry, histological and behavioral parameters, and meat lipid profiles were assessed. In the second study, the apparent digestibility of diets containing 0, 25, or 50 mg HT/kg feed was evaluated using 15 male piglets (21.5 ± 1.5 kg) through total excreta collection. Results revealed that HT influenced serum glucose and gamma-glutamyl transferase, histological inflammation, and active behaviors. HT modified lipid profiles, reduced capric, lauric, linolenic, arachidonic, cis-5,8,11,14,17-eicosapentaenoic fatty acid concentrations, and increased the nervonic acid profile. The digestibility of dry matter, organic matter, energy, and protein increased with HT use up to 50 mg/kg of feed. These findings demonstrate that HT positively impacts piglet efficiency, changing the fatty acid profile with increased nervonic acid, highlighting its potential as a dietary additive for improving nursery pig production.

## Simple Summary

Piglets are susceptible to various health and growth challenges during the nursery phase. To address these issues, two studies were conducted to explore how adding hydroxytyrosol (HT), a natural compound found in olives, to their diets might help. The first study tested how different amounts of HT affected the piglets’ growth, health, behavior, and meat fat profile. The second study investigated how well piglets could digest their feed when fed diets with HT. Results showed that HT change certain health markers and digestion, influenced meat fat composition. The findings suggest that HT could enhance piglet health and productivity, providing benefits for pigs and potentially leading to more sustainable pig farming.

## 1. Introduction

Nutrition plays an important role in the development and health of piglets, especially during the nursery phase, a period characterized by stress due to weaning and changes in diet [1]. The challenges during weaning lead to the search for feed additives that can improve the health and performance of piglets, with hydroxytyrosol being a compound with potential for this function given its antioxidant, anti-inflammatory, and antimicrobial properties [2,3].

Hydroxytyrosol (HT) is a polyphenol present mainly in the leaves and fruits of the olive tree (*Olea europaea* L.), being one of the active components of olive oil [4]. Studies in humans and other species indicate benefits of HT, suggesting its potential in promoting intestinal health and modulating the immune response [2,5]. However, the application of HT in swine feeding, particularly in nursery pigs, is still a developing field that deserves detailed investigation.

In the specialized literature related to additives in nutrition, it is observed that the inclusion of antioxidant additives in the diet of piglets is an established practice to improve intestinal health and reduce oxidative stress, as, for example, in the study carried out by researchers [6] in which they observed that the combination of vitamin E and HT improved the lipid composition of the sows’ milk and, as a result, promoted better health conditions for the piglets after weaning.

Recent studies indicate that HT can improve intestinal mucosal integrity, increase total antioxidant capacity, and reduce the incidence of enteric diseases in broilers [7]. A study conducted by researchers [8] in which they investigated the effect of HT on intestinal oxidative stress in piglets found that HT addition increased the expression of genes related to intestinal barrier integrity and reduced pro-inflammatory cytokines, such as IL-1β, IL-6, and TNF-α. Additionally, in other species, such as chickens, the use of up to 50 mg of 1-HT/kg of feed promoted changes in the breast fat profile, reducing the contents of several fat acids, and helped to protect the liver against inflammation [9]. In piglets, maternal HT addition showed significant and highly consistent effects on the fatty acid fractions of the liver and the longissimus dorsi muscle [10].

Additionally, HT is safe and non-toxic, and researchers [11] conducted a toxicological evaluation of pure HT and proposed a no-observed-adverse-effect level of 500 mg of HT/kg body weight per day, supporting the safety profile of this compound. In a subsequent study, the same team reported no mutagenic or genotoxic effects of HT in an in vitro study [12].

Therefore, the hypothesis of the present work is that the inclusion of HT in the diet of piglets in the nursery phase could enhance zootechnical performance and intestinal health, reduce oxidative stress, and improve digestibility and promote a better fat acid profile. Thus, the aim of this study was to evaluate the effects of HT addition on the performance, serum biochemistry, intestinal morphology, behavior, lipid profiles, and digestibility of piglets in the nursery phase.

## 2. Materials and Methods

### 2.1. Experiment 1

#### 2.1.1. Facilities

The experiment was conducted at the UDESC Experimental Farm—Oeste (FECEO), (27°09′ S; 52°47′ W) in the State of Santa Catarina, Brazil. The experiment was conducted in a swine nutrition research facility consisting of two experimental rooms (Room 1—designated for performance evaluation studies, and Room 2—designated for digestibility studies). For the first experiment, Room 1 was used, which was equipped with 36 pens measuring 1.2 m × 0.9 m, each with a capacity to house three piglets. The pens were equipped with a linear trough-type feeder with 3 manual filling nozzles and a nipple-type drinker with height adjustment and a minimum flow rate of 1.5 L/min and a total slatted floor made of high-resistance plastic.

The air temperature was controlled through curtain management and an automatic convective heating system. The dry bulb temperature (DBT) and relative humidity (RH) were recorded at hourly intervals using a data logger positioned at the geometric center of the experimental facility, installed 60 cm above the floor and equipped with a DHT22 probe. The external temperature was obtained from an automated climatological station (Plugfield model WS20, Curitiba, Brazil) positioned 50 m from the experimental facility. The temperatures recorded inside the experimental facility during the weeks of the experiment were close to the comfort temperature for the pigs in the respective phase (Figure S1).

#### 2.1.2. Piglets/Feeds and Treatments

For the first study, 72 male piglets were used, comprising commercial hybrids selected for high lean meat deposition from the crossing of the female of Aurora genetics (GA-2030 based on animals of the Landrace and Large White breeds) with the male terminator Agroceres (AGPIC 337), weaned at approximately 26 days of age, with an initial weight of 7.30 ± 0.52 kg, housed in 24 experimental pens. A 42-day trial period was adopted.

The nutritional levels of the diets and the nutritional compositions of the feeds were based on the values proposed by [13] (Table 1); the rations were manufactured in a commercial feed factory, holder of IN14/IN65 Brazil [14,15]. Feed was provided *ad libitum*, with manual replenishment performed several times throughout the day (07:30–17:00) to ensure continuous feed availability. No feed was offered during the night.

Seventy-two piglets were randomly assigned to one of four experimental treatments with increasing levels of HT inclusion (0, 5, 10 or 50 mg of HT/kg of feed), with 6 replicates (pen) per treatment and 3 male piglets per pen. For HT addition, commercial 1-HT^®^ hydroxytyrosol in powder form (25% hydroxytyrosol at a ≥98% purity and 75% chicory root inulin as excipient), produced by Nova Mentis Ltd., Dublin, Ireland, was used.

To incorporate HT into the treatments, approximately 1.5 kg of the basal diet was first premixed with the required amount of HT. This premix was then added to the mixer containing the remaining portion of the basal diet and thoroughly homogenized. This procedure was repeated for each HT-containing treatment.

After adding the additives, the rations were sampled and ground in a hammer mill with a 1 mm sieve, and the dry matter (DM, method 930.15), crude protein (CP, method 988.05), ashes (MM, method 942.05), and ether extract (EE, method 920.39) contents were analyzed using the methodologies described by [16].

#### 2.1.3. Zootechnical Performance

At the start and at the end of each phase, the piglets were weighed (starting at 08:00 a.m.) on a digital scale (Líder^®^ model b-150, 30 kg ± 5 g, Araçatuba, Brazil), and the feed intake was computed to calculate the average daily feed intake (ADI), average daily weight gain (ADG), and feed conversion (FCR), calculated based on the ratio between ADI and ADG. Necessary veterinary interventions were performed, when necessary, in accordance with the protocol established by the veterinary team.

#### 2.1.4. Serum Variables

Blood samples were collected (starting at 09:00 a.m.) on days 14 and 35 via puncture of the cranial vena cava, a procedure performed by a trained person (5 mL per piglet) using a 40 mm needle and vacutainer tubes. On the blood sample days, feeding started after blood collection. The piglet with an intermediate weight in the pen was selected for collection. The samples were stored in tubes containing an anticoagulant (heparin) and kept refrigerated. A fraction of the sample was used for the analysis of hematological variables (Equip Vet analyzer 3000^®^, Chongqing, China). The remaining samples were centrifuged (3500 rpm for 10 min), and the serum was stored (−20 °C) for later serum analyses.

Serum levels of total protein (PROTEI), albumin (ALBU), urea (URE), creatinine (CR), aspartate aminotransferase (AST), alanine aminotransferase (ALT), and gamma-glutamyl transferase (GGT) were evaluated using commercial analytical kits (Analisa^®^, Jakarta, Indonesia) and a semiautomatic analyzer (BioPlus-2000 c, Altamonte Springs, FL, USA). Serum globulin levels were estimated (total protein-albumin).

#### 2.1.5. Morphometric Variables and Tissue Collection

At the end of the experimental period, one piglet from each pen (6 pigs per treatment) was slaughtered in a commercial slaughterhouse following animal welfare standards, and the carcass were kept in a cold chamber (approximately 5 °C).

Muscle samples (chops ± 2 cm thick) were taken at the last rib (P2 position) 24 h pos-mortem. After collecting, the samples were frozen (−20 °C) until processing for fatty acid profile analysis.

Immediately after evisceration, jejunum samples were collected approximately 30 cm from the starting point. The jejunum samples were fixed in 10% formalin solution for at least 24 h. All samples were dehydrated, infiltrated, and embedded in paraffin following standard histological procedures. Tissue blocks were cut into 5 μm sections and stained with hematoxylin and eosin.

For intestinal morphology analysis, one slide and 20 intestinal villi per pig were evaluated at a 10× magnification (using 20× and 40× magnification to confirm alterations) using an optical microscope (Nikon Eclipse E200, São Paulo, Brazil).

In the intestinal samples, the following parameters were scored: enterocyte proliferation; inflammatory infiltration in the epithelium; inflammatory infiltration in the lamina propria; goblet cell proliferation, congestion, and sum of the variables, according to the ISI (I see inside) methodology [17], adapted for piglets.

The ISI methodology for microscopy followed [18], while the histological routine was based on research [17], adapted from the same source. The ISI method (patent pending: INPI BR 1020150036019) uses a numeric scoring system to evaluate microscopic alterations. Each alteration is assigned an impact factor (IF) ranging from 1 to 3, reflecting its effect on organ function.

#### 2.1.6. Behavioral Analysis

Behaviors were assessed using a previously tested work ethogram with the following behaviors: consuming (eating feed or drinking water); active behaviors (biting another piglet, or settling or fighting; standing still or exploring); inactive behaviors (lying down, sitting alert, or sleeping); and interaction with an enrichment object. The enrichment object was a 30 cm plastic strap attached to the side of the pen (one per pen) at the height of the piglet’s head, installed immediately before the start of observations, and replaced immediately if removed by the piglet.

The instantaneous evaluation methodology was applied with a 5 min sampling interval, performed by trained observers, according to the adapted methodology proposed by two authors [19] with two observers alternating every 5 min. Prior to the observations, the piglets were identified on their backs with non-toxic paint. Observations were carried out on three days throughout the experimental period (d 7, d 14, and d 28) totaling 108 observations per piglet. The behaviors obtained were converted into percentages for later analysis in which the effects of the observation days, treatments, and respective interactions were analyzed.

#### 2.1.7. Lipid Profile Analysis

In the muscle samples, the profile of deposited fatty acids was analyzed, and the extraction of fatty acids was carried out using the method of researchers [20]; 2.8 g of meat samples, 4.3 mL of water, 16 mL of methanol, and 8 mL of chloroform were added in a 50 mL polypropylene tube and homogenized in turrax until disintegration of the sample was completed, followed by agitation mechanics for 60 min, followed by the addition of 8 mL of chloroform and a 1.5% Na_2_SO_4_ solution to promote a biphasic system. This mixture was agitated for 2 min and then centrifuged for 15 min at 2000 rpm. The lipids obtained from the chloroform phase were subjected to fatty acid analysis.

The methylation of FA was performed using a transesterification method proposed by [21]. To the extracted lipids, 1 mL of 0.4 M KOH methanolic solution was added in a test tube and vortexed for 1 min. The samples were kept in a water bath for 10 min at the boiling point. Subsequently, they were cooled to room temperature, and 3 mL of a 1 M H_2_SO_4_ methanolic solution was added, vortexed, and kept in a water bath for 10 min. After cooling, 2 mL of hexane was added and centrifuged at 2000 rpm for 10 min. Finally, the hexane with fatty acid methyl esters (FAMEs) was subjected to chromatographic analysis.

For the determination of FAMEs, a gas chromatograph (TRACE 1310) equipped with a flame ionization detector (Thermo Scientific, Waltham, MA, USA) was used. One microliter of sample was injected in a split/splitless injector, operated in 1:10 split mode at 250 °C. Hydrogen was used as carrier gas at a constant flow of 1.5 mL/min. The separation of FAMEs was carried out in a RT 2560 chromatography column (100 m × 0.25 mm × 0.20 μm film thickness, Restek, Bellefonte, PA, USA). The temperature of the oven was programmed at 100 °C for 5 min at the start and increased to 180 °C at a rate of 8 °C/min, then increasing to 210 °C at a rate of 4 °C/min, and finally up to 240 °C, increasing by 20 °C/min, and maintained for 20 min in isothermal. The detector temperature was maintained constant at 250 °C. The FAMEs in the analytes of the samples were identified based on the retention times in comparison with those found in the FAME Mix-37 standard (P/N 47885-U, Sigma-Aldrich, St. Louis, MO, USA). The fatty acids, in mg/g, of total lipids were quantified in relation to the internal standard (IS), methyl tricosanoate (C23:0), and considering the factor FAME chain length equivalent to FID, and the ester conversion factor to the respective acid was applied according to the literature (SIMIONATO e colab., 2015 [22]).

### 2.2. Experiment 2

#### Assessment of Digestibility Coefficients

This step was carried out after Experiment 1 was fully completed, as a complementary study in which we included the 25 mg/kg level. In this trial, we used 15 male pigs, from the same farm of those described in the exp. 1, with an initial body weight of 21.5 ± 1.5 kg. The pigs (5 per treatment) were allotted to three treatments (0, 25, or 50 mg of HT/kg of feed). The experimental diet was the same as the initial diet used in experiment 1 (Table 1). The HT was incorporated following the same methodology previously described (item 2.1).

The trial was conducted in a metabolism room equipped with individual digestibility pens [23]. A 7-day adaptation period to the metabolism pens and diets, followed by 5 days of total collection (feces and urine), was adopted for the study. The feeding and total collection method, using Fe_2_O_3_ (1.5%) as a fecal marker, followed the recommendations of researchers [24,25].

The total collection was sub-sampled and dried, and the dry matter, organic matter, gross energy, and crude protein contents were analyzed using the same methodologies described in experiment 1. Subsequently, with the analyzed composition and the quantity consumed and excreted, the apparent digestibility coefficients were calculated ((ingested − excreted)/ingested × 100).

### 2.3. Statistical Analysis

For the first experiment, six replicates per treatment were used. Behavioral and performance variables were obtained from the pen average, while the other variables in this step were obtained from the piglet with an intermediate body weight in the pen. For the sequential study (digestibility experiment), five replicates/piglet per treatment were used. Initially, the data obtained were assessed for normality of errors using the Shapiro–Wilk test (α > 0.05) and transformed when necessary to meet normality requirements using Past 4 Software. Subsequently, they were analyzed based on a randomized design with HT levels as the independent variable, with the initial weight as a covariate for performance analyses. If the covariate was not significant (α > 0.05), it was removed from the model to avoid introducing statistical noise. Subsequently, significant variables (α < 0.05) or a trend toward significance (≥0.05 and α < 0.10) were subjected to the Tukey test (α < 0.05). As a complementary measure, regression analysis, in which linear and quadratic models were tested, was performed. In the case of equations with significant adjustment (α < 0.05), the coefficients of the equations were tested using the *t*-test (α < 0.05). When the quadratic fit model promoted the best fit, the equations were derived to determine the point of inflection.

## 3. Results

### 3.1. Performance

The variables average daily feed intake and average daily weight gain were not influenced (*p* > 0.05) by the HT levels studied (Table 2), the means of the FC at 14 and 28 days showed a tendency (*p* < 0.10) of a difference with the use of HT and were better adjusted with a decreasing linear equation with the increase in HT levels (*p* < 0.05), and the other FC variables were not influenced by the treatments tested (*p* > 0.05).

### 3.2. Serum Biochemistry

For the serum biochemistry at the 14-day collection (Table 3), there was no effect (*p* > 0.05) of the HT levels on the variables evaluated. In the 35-day collection, two of the variables evaluated, gamma- glutamyl transferase (GGT) and glucose (GLU), were influenced (*p* < 0.05) in response to different levels of HT. The other serum variables of the second collection (35 d) were not influenced (*p* > 0.05) by the treatments studied.

Glucose levels were significantly higher in the HT 10 treatment vs. the control treatment (*p* < 0.05), while no differences were observed among the others (*p* > 0.05). The regression analyses show the best fit with the quadratic regression model (*p* < 0.05) with a maximum point estimated point at 26.5 mg of HT/kg of feed. GGT activity was higher in the HT 5 treatment compared to HT 10 (*p* < 0.05), while no differences (*p* > 0.05) were observed among the other treatments, and the data were not adjusted to the regression models studied (*p* > 0.05).

### 3.3. Intestinal Histology

Only the intestinal variable inflammatory infiltration in the lamina propria (INFLP) showed differences (*p* < 0.05), but this variable did not differ among treatments according to Tukey’s test (*p* > 0.05) (Table 4), and the INFLP was best adjusted (*p* < 0.05) with a quadratic equation with an inflection point estimated at 23.8 mg of HT/kg of feed. The sum of the evaluated scores trended (*p* < 0.10) for an effect of treatments, and the other variables did not differ (*p* > 0.05) among the treatments studied.

### 3.4. Behaviors

Behavior related to energy-demanding activities (ACTBEH) was influenced by treatments (*p* < 0.05) with the 10 mg HT/kg of feed level higher than the 5 mg HT/kg of feed treatment. The treatments with 0, 10, and 50 mg of HT/kg of feed did not show significant differences (*p* > 0.05), according to Tukey’s test, and the regression models tested were not significant (*p* > 0.05). The other behavior variables were not influenced by the treatments (Table 5).

### 3.5. Lipid Concentration

The arachidonic and total ω3 variables did not differ among treatments according to Tukey’s test (*p* > 0.05) (Table 6). The levels of capric, cis-5,8,11,14,17-eicosapentaenoic, and nervonic acids in the 50 mg HT/kg treatment were higher than the control (without HT) treatment (*p* < 0.05), and lignoceric at level 10 mg/HT was higher than in the control group (*p* < 0.05).

Additionally, increasing doses of HT promoted a linear reduction in the levels of capric, lauric, linolenic, arachidonic, and cis-5,8,11,14,17-eicosapentaenoic and promoted a linear increase in nervonic acid levels (*p* < 0.05). The concentration of lignoceric acid (C24:0) was best adjusted by the quadratic model (*p* < 0.05) with an estimated maximum point at 25.8 mg of HT/kg of feed (Table 6).

### 3.6. Apparent Digestibility Coefficients

The apparent digestibility coefficients of organic matter and the apparent metabolic utilization of gross energy were higher in the 50 mg/kg of HT treatment than in the control treatment (*p* < 0.05), and 25 mg/kg of HT did not differ from the other treatments (Table 7). The means of apparent metabolic utilization of crude protein did not differ among treatments according to Tukey’s test (*p* > 0.05). The means of the apparent digestibility coefficient of dry matter and gross energy indicated a trend (*p* < 0.10) with the treatments evaluated.

Digestibility variables that showed differences (*p* < 0.05) or a trend (*p* < 0.10) were best fitted by a linear model with an increasing response (*p* < 0.05).

## 4. Discussion

### 4.1. Performance

The results indicate that HT addition up to 50 mg of HT/kg of feed did not affect the average daily feed intake and average daily weight gain of piglets, but there was a trend towards improved feed conversion with linear improvement with its inclusion. This suggests that HT may be a beneficial additive in swine diets, particularly to optimize feed conversion.

Recent studies indicate that HT may have neutral or positive effects on feed intake depending on the concentration used [8]. According to the literature [26], the addition of antioxidant such as *Caesalpinia sappan*, green tea, grape seed extracts, and essential oil–cyclodextrin complexes to swine diets can improve feed stability and reduce lipid oxidation, potentially enhancing palatability and increasing feed intake. In the current study, the feeds were produced in the week prior to their use and were also enriched with antioxidant additives (Table 1), which may have limited the effects of HT on the preservation of feed components. Nevertheless, the lack of a significant effect in the present study suggests that the HT levels used were within a range that does not alter the feed intake of piglets.

Thus, the observed effects of a tendency for improvements in FC may be associated with the antioxidant and anti-inflammatory effects of HT on piglets. In a study conducted by researchers [27], piglets fed a commercial blend of antioxidants, either LOXIDAN VD100 at 150 g/t (containing butylated hydroxytoluene, propyl gallate, and citric acid) or LOXIDAN E Ros at 300 g/t (containing α, β, γ, and δ tocopherol extracts), showed improved feed conversion and greater body weight gain compared to the negative control group. The authors associated these results with a reduction in oxidative stress, which promoted greater piglet metabolic efficiency.

The present study corroborates these findings by indicating an improvement in feed conversion with the inclusion of HT, which is probably associated with the better intestinal health and better metabolic efficiency of piglets. In another study with olive derivatives (fermented mixture of olive stone residues and *Lathyrus clymenum*) supplemented in the diet of weaned piglets, improvements were observed in serum antioxidant markers, including increased concentrations of glutathione and catalase, and a reduction in thiobarbituric acid reactive species. The authors associated these results with enhanced health and oxidative status in the piglets [28]. The inclusion of an encapsulated mixture of organic acids (fumaric, citric, and benzoic) and essential oils (thymol, eugenol, cinnamaldehyde, and carvacrol) improved the intestinal health of piglets [29]. Based on this study, which demonstrated the efficacy of such a combination, it can be inferred that the addition of HT may enhance these beneficial effects, due to its anti-inflammatory and antioxidant properties.

### 4.2. Serum Biochemistry

The lower activity of the GGT variable in the treatment with 10 mg of HT/kg of feed may be associated with the hepatoprotective effect of HT at this dosage. GGT is an important marker of liver function [9,30] and oxidative stress [8,31], and low levels may be associated with liver health.

Glucose levels were best fitted with a quadratic regression model, indicating a peak point at 26.5 mg of HT/kg of feed, suggesting that there is an ideal dosage of HT to optimize glucose levels. The observed increase in glucose may be associated with better gut health, which leads to the better absorption of nutrients from the diet.

A limitation of our results is the absence of differences between the treatments with 5 and 50 mg of HT/kg of feed and the treatment without HT inclusion, a finding that requires further studies to confirm the previously proposed hypothesis.

However, we highlight that although HT levels influenced GGT and glucose, the results obtained are within the range considered adequate for the aforementioned variables for piglets, which vary from 0 to 82 U/L for GGT and 77.5 to 154.9 mg/dL for glucose, respectively [32].

### 4.3. Intestinal Histology

According regression analysis, the use of HT at doses close to 25 mg/kg of feed reduced inflammatory infiltration in the lamina propria, and for concentrations close to 50 mg of HT/kg of feed, the benefit was not maintained. Thus, although in the present study, the effects of HT on cell proliferation and inflammatory infiltration in the epithelium were not significant, the reduction in inflammatory infiltration in the lamina propria is consistent with the literature [33], which points to the anti-inflammatory benefits of antioxidants for the intestinal health of pigs [34,35].

The results of the sum of the histological variables evaluated (PROENT, INFEP INFLP, GLOB, and CONG) indicate a trend in response to the administration of different concentrations of HT with a reduction in intermediate doses, which suggests that the additive may have a positive effect on the histological characteristics of the intestines of pigs.

The trend observed in the present study is consistent with recent research investigating the effects of other antioxidant compounds on intestinal health in pigs, such as in the studies by researchers [6,36], who observed that addition of vitamin E or a combination of vitamin E with HT (1.5 mg of HT/kg of feed) improved the oxidative status, intestinal homeostasis, and intestinal health of sows and piglets. These authors reported an improvement in intestinal integrity, corroborating the idea that antioxidant compounds may have beneficial effects on intestinal health in pigs.

Furthermore, in a study conducted by researchers [37], which investigated the effects of antioxidant supplementation (polyphenol ellagic acid at 500 ppm) on the intestinal health and inflammatory response of young piglets, they found that the supplementation reduced the inflammatory response and promoted the growth and intestinal health of piglets.

However, similar to what was discussed for the variables that showed differences in Section 4.2, these results require further studies, given the lack of differences among treatments with 5 and 50 mg of HT/kg of feed and the treatment without HT inclusion, according to Tukey’s test.

### 4.4. Behavior

The increase in ACTBEH behaviors of piglets with the administration of 10 mg of HT/kg of feed suggests that HT may have a positive effect on the frequency of activities that involve energy expenditure. Existing literature reinforces the idea that HT has neuroprotective capacity and can improve neurophysiological health [38].

The anti-inflammatory properties of HT are described by several authors [39]; when evaluating the use of HT for rats with inflammation (rheumatoid arthritis), a significant impact of HT on inflammatory processes was found. Researchers [33] found a reduction in inflammatory biomarkers in rats with ulcerative colitis and [40] demonstrated the anti-inflammatory and anti-aging effects of HT on human dermal fibroblast cells.

Inflammatory processes can lead to changes in behavioral patterns in pigs, since cytokines produced at the beginning of the inflammatory process can cause a reduction in general activities and a loss of interest in social activities [41]. Similar results occur in dogs and cats [40], with changes in behavioral patterns, in which the authors argue that the change in motivational state allows the individual to conserve energy and stay away from danger to recover.

These results are consistent with the results of the present study, in which the administration of HT at doses of 10 mg of HT/kg of feed was associated with an increase in piglet activity. But the similar results in active behavior among treatments with 5 and 50 mg of HT/kg of feed and the treatment without HT inclusion, according to Tukey’s test, require further studies.

### 4.5. Lipid Profile of Meat

In the present study, lower fatty acids such as capric (C10:0), lauric (C12:0), arachidonic (C20:4n6), and eicosapentaenoic (C20:5n3) were observed, which suggests that the use of HT in feeds may affect the metabolism of fatty acids in the body and consequently their profile in meat, possibly regulating the enzymes involved in the production and modification of these compounds. A study by researchers [42] indicated that antioxidants can reduce lipid oxidation in meat, thus influencing the deposition of fatty acids.

HT, known for its antioxidant properties, may have contributed to the reduction in short-chain saturated fatty acids, possibly through protective mechanisms against lipid oxidation. The increase in monounsaturated fatty acids such as nervonic acid (C24:1n9), although not an essential fatty acid, is particularly interesting given the positive implications that this fatty acid has for brain health and neurological diseases [43].

Nervonic acid is the most common long-chain monounsaturated fatty acid found in the white matter of the brain [44]. In a study by researchers [45], the authors highlighted the role of nervonic acid in the composition of myelin as crucial for the functioning of nerve cells. The same article also emphasizes the actions of nervonic acid in metabolic processes, particularly in the regulation of lipid metabolism and potential beneficial effects on neurodegenerative conditions.

The fatty acids that, through the action of elongases, can be precursors of nervonic acid are palmitic acid, stearic acid, oleic acid, and eicosenoic acid [46], in addition to erucic acid. However, there was no change in the profile of the precursor fatty acids of nervonic acid, which leads to the hypothesis that the increase obtained in the concentration of nervonic acid may have occurred due to greater intestinal absorption or greater synthesis by the intestinal microbiota. The digestion and absorption of lipids is dependent on bile juice and is being studied to evaluate intestinal oxidative stress in young piglets, and [8] found that oxidative stress can cause disorders in bile acid metabolism with lower levels of bile acids such as hyocholic acid, hyodeoxycholic acid, and tauroursodeoxycholic acid, which were partially restored by the inclusion of HT. However, this hypothesis needs to be further elucidated in other studies.

The observed effects of HT on the reduction or tendency to reduce specific saturated fatty acids and some PUFAs are consistent with other studies investigating natural antioxidants in diets for growing-to-finishing pigs [47]; in this work, the authors demonstrated that dietary additives with plant extracts can improve pork quality by positively influencing the fatty acid profile and oxidative stability. This study supports the hypothesis that additives, such as HT, can modify the fatty acid composition in meat, possibly due to their ability to influence lipid metabolism and fatty acid synthesis.

In the present study, the ω6/ω3 ratio was not affected by the inclusion of HT, although there was an effect of HT levels on the sum of ∑ω3, probably associated with the reduction of C20:5n3, with a linear trend of reduction.

### 4.6. Digestibility Coefficients

The results obtained with improvement in the apparent coefficients of dry matter (DM), gross energy (GE), and apparent metabolic utilization of crude protein (CP) indicate a positive effect of HT on the digestion of nutrients in the feed and confirm the results obtained in the previous stage with a tendency for improvement in the feed conversion.

HT has antioxidant properties at the intestinal level that may be beneficial for intestinal morphology, with reduced expression of inflammatory cytokines and partial restoration of the bile acid composition [8]. These characteristics, combined, can favor the best digestibility coefficients obtained.

In a study conducted by researchers [48] to evaluate the digestibility of diets with the inclusion of olive leaves, a reduction in protein and fat digestibility was observed. The authors attributed these results to the fiber content of the leaves, which is negatively correlated with digestibility. In a study that evaluated the effects of the addition of olive leaf extract (96 mg/day of HT in the treatment with the highest inclusion), there were no effects on the digestibility of dry matter, energy, and protein, with a reduction in the retention of Fe and K [3]. However, in the present study, the piglets used had a higher body weight than in the previously reported study. Nevertheless, we emphasize that the HT used in the current study was obtained via biotechnological synthesis, presenting high purity, which may be associated with the difference between the results obtained. It is worth mentioning that our results with biotechnologically produced HT are supported by other authors, such as [49], who in a review study, discussed that polyphenols can, during the stress of weaning piglets, favor nutrient absorption and digestion.

## 5. Conclusions

The inclusion of HT in pigs’ diet influenced the serum biochemistry, intestinal histology, and active behavior of pigs. HT inclusion also affected the lipid profile of meat by reducing some of the saturated fatty acids and increasing the monounsaturated fatty acid nervonic acid. HT inclusion also showed a tendency for improvement in feed conversion up to a dose of 50 mg of HT/kg of feed. Additionally, it improves the digestibility coefficient of dry matter, energy, and protein metabolism. The inclusion of HT as a dietary additive for weaned piglets showed potential to enhance performance, improve the overall health status, and support better feed nutrient utilization.

## Figures and Tables

**Table 1:** Composition of the experimental diets used during the experiment (exp. 1 and 2).

Ingredients, kg/ton *	Pre-I (0–7 d)	Pre-II (8–14 d)	Initial (15–42 d)
Ground Corn	9.800	41.145	63.963
Pre-gelatinized Corn	25.600	10.000	0.000
Soybean Meal	14.350	19.800	25.850
Micronized Soy	8.050	6.000	2.490
Soy Protein Concentrate	4.000	1.500	0.000
Whey	20.000	10.000	0.000
Cookie waste	6.000	3.000	0.000
Dehydrated Egg Flour	4.000	2.000	0.000
Sugar	4.000	2.500	0.984
Calcitic Limestone	0.850	0.600	0.780
Dicalcium Phosphate	0.900	1.020	1.259
Bewi^®^	0.000	0.000	2.840
Sodium Bicarbonate	0.075	0.100	0.380
Refined Salt	0.100	0.250	0.230
L-Lysine 98.5%	0.480	0.465	0.330
DL-Methionine 99%	0.210	0.180	0.108
L-Threonine 98.5%	0.240	0.220	0.125
L-Tryptophan 98%	0.045	0.045	0.018
L-Isoleucine 97.5%	0.000	0.005	0.000
L-Valine 98%	0.075	0.075	0.000
Hostazym X100^®^	0.010	0.010	0.010
OptiPhos Plus^®^	0.003	0.005	0.003
Sucram C 150^®^	0.020	0.020	0.020
Banox^®^	0.010	0.010	0.010
Zinc Oxide	0.175	0.125	0.050
Inert^®^	0.608	0.525	0.150
Vitamin mineral supplement	0.400 ^1^	0.400 ^2^	0.400 ^3^
Calculated composition, (as feed base) **			
Calcium, %	0.80	0.66	0.71
Available Phosphorus, %	0.39	0.35	0.33
Metabolizable Energy, Mcal/kg	3.49	3.37	3.34
Crude Protein, %	20.3	19.3	18.0
Digestible Lysine, %	1.43	1.29	1.07
Digestible Methionine + Cystine, %	0.80	0.73	0.61
Digestible Tryptophan, %	0.28	0.25	0.21
Digestible Threonine, %	0.96	0.86	0.69
Digestible Valine, %	0.97	0.889	0.75
Digestible Isoleucine, %	0.83	0.75	0.67

* Bewi^®^—palm fat and lecithin; Hostazym^®^—enzyme to increases nutrient digestibility (mainly energy, fat, and protein); OptiPhos^®^—commercial phytase for use in animal nutrition; Sucram—palatability enhancer to improve feed intake; Banox^®^—antioxidant additive; Inert—inert material; ** Values calculated based on the nutritional composition proposed by [13]; ^1^—Minimum guaranteed levels per g of product: Crude Fiber (CF) 60 g; Ca 7 g; P 5 g; Na 1 g; Cu 125 g; Fe 199 g; I 1.2 g; Mn 52 g; Se 0.4 g; Zn 104 g; L-Lys 10 g; L-Val 6000 g; DL-Met 3000 g; L-Thr 7000 g; L-Trp 2000 g and Phytase 1000 FTU; Vitamins: Folic Acid 2 g; Pantothenic Acid 26 g; Biotin 0.30 g; Choline 1.145 g; Niacin 39 g; A 15.720 IU; B1 2.7 g; B12 41.9 g; B2 5.5 g; B6 4.1 g; D3 3.140 g; E 167 IU; K3 5.4 g; ^2^—Minimum guaranteed levels per g of product: CF 75 g; Ca 6 g; P 5 g; Na 2 g; Cu 123 g; Fe 195 g; I 1.2 g; Mn 51 g; Se 0.4 g; Zn 102 g; L-Lys 10 g; L-Isoleucine 5.000 g; L-Trp 2.000 g; L-Val 6000 g; DL-Met 3000 g; L-Thr 7000 g and Phytase 1000 FTU; Vitamins: Folic Acid 2 g; Pantothenic Acid 25 g; Biotin 0.30 g; Choline 1.270 g; Niacin 38 g; A 15.421 IU; B1 2.6 g; B12 41.1 g; B2 5.4 g; B6 4 g; D3 3.080 IU; E 164 IU; K3 5.3 g; ^3^—Minimum guaranteed levels per g of product: CF 100 g; Ca 6000 g; P 5000 g; Na 2 g; Cu 120 g; Fe 190 g; I 1.2 g; Mn 50 g; Se 0.4 g; Zn 100 g; Lys 10 g; L-Trp 2000 g; DL-Met 3000 g; L-Thr 7000 g; Phytase 500 FTU and Xylanase 1500 FTU; Vitamins: Folic Acid 2 g; Pantothenic Acid 25 g; Biotin 0.30 g; Choline 1400 g; Niacin 37 g; A 15,037 IU; B1 2.6 g; B12 40 g; B2 5.2 g; B6 3.9 g; D3 3007 IU; E 160 IU; K3 5.2 g.

**Table 2:** Zootechnical performance of piglets fed diets with increasing inclusion levels of HT per kg of feed (Exp. 1).

	HT Inclusion Levels, mg/kg of Feed				Qualitative Analysis		Regression Analysis	
	0	5	10	50	*p*=	SEM	Linear	Quadratic
Initial BW, kg	7.30	7.29	7.31	7.30	1.000	0.103	NA	NA
Final BW, kg	26.43	26.25	26.8	25.78	0.873	0.381	NA	NA
	Average daily feed intake (DFI), kg/day							
DFI 0–7 days	0.277	0.269	0.310	0.327	0.477	0.014	NA	NA
DFI 0–14 days	0.376	0.382	0.400	0.403	0.626	0.008	NA	NA
DFI 0–28 days	0.559	0.569	0.570	0.553	0.914	0.009	NA	NA
DFI 0–42 days	0.773	0.731	0.774	0.742	0.626	0.014	NA	NA
	Average daily weight gain (DWG), kg/day							
DWG 0–7 days	0.139	0.152	0.174	0.133	0.321	0.008	NA	NA
DWG 0–14 days	0.254	0.266	0.259	0.267	0.814	0.005	NA	NA
DWG 0–28 days	0.365	0.369	0.368	0.364	0.993	0.007	NA	NA
DWG 0–42 days	0.456	0.451	0.464	0.440	0.795	0.008	NA	NA
	Feed conversion (FC), kg/kg *							
FC 0–7 days	1.854	1.790	1.789	1.821	0.988	0.071	NA	NA
FC 0–14 days	1.446 a	1.441 ab	1.495 a	1.323 b	0.051	0.021	0.028	0.041 *
FC 0–28 days	1.535	1.544	1.545	1.468	0.085	0.013	0.023	0.34 *
FC 0–42 days	1.693	1.623	1.708	1.664	0.264	0.024	NA	NA

HT hydroxytyrosol mg/kg of feed: hydroxytyrosol in milligrams or kilograms of feed; performance in kilograms; NA: not analyzed. * one or more of the coefficients were not significant, *p* > 0.05, in the *T* test. Note: Means followed by different letters differ based on Tukey’s test (*p* < 0.05); *p*: probability value; SEM: standard error of the mean.

**Table 3:** Serum biochemistry of nursery piglets fed with hydroxytyrosol—Experiment 1 (evaluated on days 14 and 35).

	HT Inclusion Levels, mg/kg of Feed				Qualitative Analysis		Regression Analysis	
Variables	0	5	10	50	*p*=	SEM	Linear	Quadratic
	Serum biochemistry as d 14							
ALB	2.31	2.44	2.38	2.25	0.658	0.054	NA	NA
CHOL	79.82	75.30	77.55	69.38	0.691	3.258	NA	NA
FERRI	108.98	79.40	105.58	109.10	0.842	11.586	NA	NA
GGT	32.48	39.23	26.20	33.17	0.127	1.952	NA	NA
GLU	114.17	104.67	107.83	111.33	0.690	2.841	NA	NA
PCR	28.32	28.35	28.22	28.02	0.144	0.056	NA	NA
TP	4.53	4.53	4.72	4.33	0.303	0.072	NA	NA
AST	58.97	78.13	77.17	56.48	0.366	5.634	NA	NA
ALT	60.28	69.88	61.95	69.13	0.945	4.519	NA	NA
UREA	9.40	6.87	8.53	7.85	0.480	0.780	NA	NA
TG	69.42	41.20	52.43	45.42	0.930	7.930	NA	NA
	Serum biochemistry as d 35							
ALB	2.39	2.52	2.53	2.38	0.568	0.046	NA	NA
CHOL	89.17	90.83	97.28	94.70	0.344	1.717	NA	NA
FERRI	96.15	73.65	100.93	95.68	0.662	8.063	NA	NA
GGT	35.58 ab	44.03 a	30.27 b	41.45 ab	0.018	1.786	0.347	0.369
GLU	114.83 b	120.50 ab	134.67 a	119.83 ab	0.035	2.624	0.930	0.019
PCR	28.25	28.08	28.12	28.15	0.653	0.047	NA	NA
TP	4.92	4.90	5.00	4.92	0.925	0.054	NA	NA
AST	45.65	45.42	47.98	49.40	0.833	1.762	NA	NA
ALT	59.00	65.73	64.95	84.60	0.125	4.150	NA	NA
UREA	15.90	16.07	11.62	13.93	0.331	0.958	NA	NA
TG	55.63	53.58	57.33	53.53	0.956	2.637	NA	NA

HT (g/ton): hydroxytyrosol; ALB (g/dL): albumin; CHOL (mg/dL): cholesterol; FERRI (ug/L): ferritin; GGT (U/L): gamma-glutamyl transferase; GLU (mg/dL): glucose; PCR (mg/L): C-reactive protein; TP (g/dL): total protein; AST (U/L): aspartate; ALT (U/L): alanine aminotransferase; URE (mg/dL): urea; TG (mg/dL): triglycerides. NA: not analyzed. Note: Means followed by different letters differ based on Tukey’s test (*p* < 0.05). Equations: glucose = 113.2256 + 2.39566 (HT) − 0.045252 (HT)^2^ (R^2^ = 0.248) (D’ = 26.47 mg/kg); *p*: probability value; SEM: standard error of the mean.

**Table 4:** Intestinal histology analysis of nursery piglets fed with hydroxytyrosol (Exp. 1).

	HT Inclusion Levels, mg/kg of Feed				Qualitative Analysis		Regression Analysis	
Items	0	5	10	50	*p*=	SEM	Linear	Quadratic
PROENT	0.70	0.65	0.79	0.75	0.347	0.029	NA	NA
INFEP	0.42	0.29	0.47	0.44	0.487	0.042	NA	NA
INFLP	1.57	1.20	1.18	1.65	0.026	0.073	0.106	0.014
GLOB	0.10	0.05	0.12	0.08	0.870	0.025	NA	NA
CONG	0.43	0.18	0.28	0.23	0.355	0.051	NA	NA
SUM	3.22	2.38	2.84	3.16	0.056	0.125	0.332	0.240

HT: hydroxytyrosol; PROENT: enterocyte proliferation; INFEP: inflammatory infiltration in the epithelium; INFLP: inflammatory infiltration in the lamina propria; GLOB: goblets cells; CONG: congestion; SUM: sum of the previous variables; NA: not analyzed. Note: Means followed by different letters differ based on Tukey’s test (*p* < 0.05). INFLP = 1.522231 − 0.04995 (HT) + 0.001051 (HT)^2^ (R^2^ = 0.270) (D’ = 23.77 mg HT/kg of feed); *p*: probability value; SEM: standard error of the mean.

**Table 5:** Behaviors of nursery piglets fed with hydroxytyrosol (Exp. 1).

	HT Inclusion Levels, mg/kg of Feed				Qualitative Analysis		Regression Analysis	
Behaviors	0	5	10	50	Treat.	SEM	Linear	Quadratic
FEEDWAT	32.8	26.9	27.7	28.3	0.101	0.993	NA	NA
ACTBEH	12.7 ab	9.6 b	14.0 a	11.9 ab	0.031	0.630	0.693	0.631
INACTBEH	23.8	30.9	27.5	29.8	0.303	1.453	NA	NA
INTERAC	30.7	32.6	30.8	30.0	0.720	1.011	NA	NA

HT: hydroxytyrosol; FEEDWAT: eating feed or drinking water; ACTBEH: biting another piglet or settling or fighting; standing still or exploring; INACTBEH: lying down, sitting alert, or sleeping; INTERAC: interacting with an enrichment object; Treat: treatment; NA: not analyzed. Note: Mean values followed by different letters differ according to Tukey’s test (*p* < 0.05); *p*: probability value; SEM: standard error of the mean.

**Table 6:** Fatty acid profiles of meat of nursery piglets fed with hydroxytyrosol (Exp. 1).

	HT Inclusion Levels, mg/kg of Feed				Qualitative Analysis		Regression Analysis	
	0	5	10	50	*p*=	SEM	Linear	Quadratic
Fat extracted, %	1.48	1.41	1.47	1.58	0.516	0.041	NA	NA
Capric	0.198 a	0.253 a	0.079 ab	0.022 b	0.004	0.036	0.040	0.096
Undecanoic	0.27	0.21	0.125	0.123	0.357	0.035	NA	NA
Lauric	0.266	0.329	0.270	0.079	0.065	0.036	0.010	0.033 *
Myristic	4.896	4.536	4.74	4.842	0.951	0.221	NA	NA
Pentadecanoic	0.992	0.967	0.883	0.846	0.507	0.038	NA	NA
Palmitic	151.1	138.8	157.6	139.3	0.426	4.681	NA	NA
Palmitoleic	12.12	10.78	12.95	10.43	0.448	0.607	NA	NA
Heptadecanoic	2.96	3.08	2.67	2.65	0.832	0.157	NA	NA
cis-10-Heptadecenoic	1.635	1.637	1.835	1.768	0.692	0.07	NA	NA
Stearic	86.88	79.73	89.227	78.61	0.363	2.491	NA	NA
Oleic	164.9	143.6	175.2	147.6	0.173	5.763	NA	NA
Linoleic	111.3	99.7	107.96	94.0	0.337	3.625	NA	NA
Arachidic	0.956	0.913	1.087	0.889	0.200	0.035	NA	NA
Linolenic	1.128	1.132	1.075	0.764	0.069	0.061	0.009	0.036 *
cis-11-Eicosenoic	2.607	2.275	2.845	2.329	0.082	0.090	0.330	0.391
a- Linolenic	2.806	2.623	2.732	2.351	0.593	0.121	NA	NA
cis-11,14-Eicosadienoic	3.308	2.734	3.137	2.846	0.119	0.094	NA	NA
Behenic	0.535	0.683	0.686	0.575	0.201	0.031	NA	NA
cis-8,11,14-Eicosatrienoic	3.202	2.853	3.07	2.728	0.324	0.097	NA	NA
Erucic	0.414	0.552	0.265	0.377	0.537	0.067	NA	NA
Arachidonic	28.69	25.06	26.59	21.08	0.041	1.008	0.008	0.029 *
cis-13,16-Docosadienoic	0.327	0.263	0.215	0.176	0.114	0.026	NA	NA
Lignoceric	0.52 b	0.72 ab	0.77 a	0.58 ab	0.029	0.035	0.569	0.015
cis-5,8,11,14,17-Eicosapentaenoic	0.59 a	0.51 ab	0.46 ab	0.38 b	0.028	0.027	0.006	0.009 *
Nervonic	0.582 b	0.733 b	0.743 b	1.214 a	<0.001	0.061	<0.001	<0.001 *
cis-4,7,10,13,16,19-Docosahexaenoic	1.056	0.893	0.862	0.766	0.169	0.047	NA	NA
∑ SFA	249.55	230.24	258.17	215.53	0.263	8.239	NA	NA
∑ UFA	334.6	296.9	339.9	301.9	0.298	9.811	NA	NA
∑ MUFA	182.2	159.5	193.8	176.6	0.356	6.738	NA	NA
∑ PUFA	152.4	137.4	146.1	125.4	0.277	5.097	NA	NA
UFA/SFA	1.34	1.30	1.31	1.46	0.633	0.048	NA	NA
∑ω6	144.3	128.8	138.7	118.8	0.235	4.675	NA	NA
∑ω3	4.45	3.53	4.06	3.49	0.038	0.143	0.065	0.134
ω6/ω3	32.57	32.42	34.50	34.18	0.508	0.592	NA	NA

Capric (C10:0); Undecanoic (C11:0); Lauric (C12:0); Myristic (C14:0); Pentadecanoic (C15:0); Palmitic (C16:0); Palmitoleic (C16:1); Heptadecanoic (C17:0); Heptadecenoic (C17:1); Stearic (C18:0); nc Oleic (C18:1n9c); nc Linoleic (C18:2n6c); Arachidic (C20:0); Linolenic (C18:3n6); cis-11-Eicosenoic (C20:1n9); a-Linolenic (C18:3n3); cis-11,14-Eicosadienoic (C20:2); Behenic (C22:0); cis-8,11,14-Eicosatrienoic (C20:3n6); Erucic (C22:1n9); Arachidonic (C20:4n6); cis-13,16-Docosadienoic (C22:2); Lignoceric (C24:0); Nervonic (C24:1n9); cis-4,7,10,13,16,19-Docosahexaenoic (C22:6n3); * one or more of the coefficients were not significant, *p* > 0.05, in the *T* test. ∑ UFA: unsaturated fatty acids; ∑ SFA Saturated fatty acids; ∑ Monounsaturated MUFA fatty acids; ∑ Polyunsaturated PUFA fatty acids. Equations: C10:0 (Capric) = 0.198205 − 0.00371 (HT) (R^2^ = 0.140); C12:0 (Lauric) = 0.308656 − 0.00448 (HT) (R^2^ = 0.232); C18:3n6 (Linolenic) = 1.148986 − 0.00765 (HT) (R^2^ = 0.237); C20:4n6 (Arachidonic) = 27.46429 − 0.12409 (HT) (R^2^ = 0.249); C24:0 (Lignoceric) = 0.538454 + 0.031073 (HT) − 0.0006035 (HT)^2^ (R^2^ = 0.264) (D’ = 25.8 mg of HT/kg of feed); C20:5n3 (cis-5,8,11,14,17-Eicosapentaenoic) = 0.541572 − 0.00351 (HT) (R^2^ = 0.263); C24:1n9 (Nervonic) = 0.624775 + 0.011869 (HT) (R^2^ = 0.623). Note: Mean values followed by different letters differ according to Tukey’s test (*p* < 0.05); *p*: probability value; SEM: standard error of the mean.

**Table 7:** Apparent digestibility coefficient (ADC) and apparent metabolic utilization (AMU) of piglets receiving different levels of HT in the diet (Exp. 2).

	HT Inclusion Levels, mg/kg of Feed			Qualitative Analysis		Regression Analysis	
	0	25	50	*p*=	SEM	Linear	Quadratic
ADC of DM, %	87.28	88.20	89.19	0.058	0.332	0.007	0.058
ADC of OM, %	88.69 b	89.83 ab	90.56 a	0.047	0.317	0.006	0.047 *
ADC of GE, %	85.29	86.29	87.61	0.059	0.409	0.008	0.059
AMU of GE, %	83.66 b	84.92 ab	86.69 a	0.011	0.452	0.001	0.011 *
ADC of CP, %	88.42	89.76	89.76	0.376	0.416	0.115	0.376
AMU of CP, %	75.74 a	76.89 a	80.17 a	0.048	0.799	0.008	0.048 *

* One or more coefficients of the equation were not significant based on the *T*-test (α > 0.05); ADC of DM (dry matter) = 87.272 + 0.00952HT (R^2^ = 0.40); ADC of MO (organic matter) = 88.767 + 0.0374 HT (R^2^ = 0.37); ADC of GE (gross energy) = 85.228 + 0.0117 HT (R^2^ = 0.40); AMC of GE (gross energy) = 83.561 + 0.0606 HT (R^2^ = 0.52); ADC of CP (crude protein); AMC CP (crude protein) = 75.312 + 0.0886 HT (R^2^ = 0.34); *p*: probability value; Note: Mean values followed by different letters differ according to Tukey’s test (*p* < 0.05); SEM: standard error of the mean.

## Data Availability

Data are contained within the article and Supplementary Materials.

## Supplementary Materials

The following supporting information can be downloaded at: https://www.mdpi.com/article/10.3390/ani15152268/s1, Figure S1: Environmental variables recorded during the experimental period.

## References

[1] Wei X., Tsai T., Howe S., Zhao J. (2021). Weaning induced gut dysfunction and nutritional interventions in nursery pigs: A partial review. Animals.

[2] Han H., Zhong R., Zhou Y., Xiong B., Chen L., Jiang Y., Liu L., Sun H., Tan J., Tao F. (2022). Hydroxytyrosol benefits boar semen quality via improving gut microbiota and blood metabolome. Front. Nutr..

[3] Leskovec J., Levart A., Salobir J., Rezar V. (2018). Do olive polyphenols negatively affect nutrient digestibility in pigs?. J. Central Eur. Agric..

[4] Cavalheiro C.V., Rosso V.D., Paulus E., Cichoski A.J., Wagner R., de Menezes C.R., Barin J.S. (2014). Composição química de folhas de oliveira (*Olea europaea* L.) da região de Caçapava do Sul, RS. Ciênc. Rural.

[5] Romani A., Ieri F., Urciuoli S., Noce A., Marrone G., Nediani C., Bernini R. (2019). Health effects of phenolic compounds found in extra-virgin olive oil, by-products, and leaf of *Olea europaea* L.. Nutrients.

[6] Laviano H.D., Gómez G., Escudero R., Nuñez Y., García-Casco J.M., Muñoz M., Heras-Molina A., López-Bote C., González-Bulnes A., Óvilo C. (2023). Maternal supplementation of vitamin E or its combination with hydroxytyrosol increases the gut health and short chain fatty acids of piglets at weaning. Antioxidants.

[7] Dias K.M.M., Oliveira C.H., Calderano A.A., Rostagno H.S., Gomes K.M., O’connor K.E., Davis R., Walsh M., Britton J., Altieri E.A. (2024). Dietary Hydroxytyrosol Supplementation on Growth Performance, Gut Morphometry, and Oxidative and Inflammatory Status in LPS-Challenged Broilers. Animals.

[8] Wen X., Wan F., Zhong R., Chen L., Zhang H. (2024). Hydroxytyrosol alleviates intestinal oxidative stress by regulating bile acid metabolism in a piglet model. Int. J. Mol. Sci..

[9] Dias K.M.M., Oliveira C.H., Calderano A.A., Rostagno H.S., O’connor K.E., Davis R., Walsh M., Britton J., Altieri E.A., Albino L.F.T. (2023). Effects of hydroxytyrosol supplementation on performance, fat and blood parameters of broiler chickens. Animals.

[10] Garcia-Contreras C., Vazquez-Gomez M., Pardo Z., Heras-Molina A., Pesantez J.L., Encinas T., Torres-Rovira L., Astiz S., Nieto R., Ovilo C. (2019). Polyphenols and IUGR pregnancies: Effects of maternal hydroxytyrosol supplementation on hepatic fat accretion and energy and fatty acids profile of fetal tissues. Nutrients.

[11] Auñon-Calles D., Canut L., Visioli F. (2013). Toxicological evaluation of pure hydroxytyrosol. Food Chem. Toxicol..

[12] Auñon-Calles D., Giordano E., Bohnenberger S., Visioli F. (2013). Hydroxytyrosol is not genotoxic in vitro. Pharmacol. Res..

[13] Rostagno H.S., Albino L.F.T., Hannas M.I., Donzele J.L., Sakomura N.K., Perazzo F.G., Saraiva A., Teixeira M.L., Rodrigues P.B., Oliveira R.F. (2017). Tabelas Brasileiras Para Aves e Suínos.

[14] (2006). Imprensa Nacional. No. 65.

[15] (2016). Imprensa Nacional. No. 14.

[16] AOAC (1990). Official Methods of Analysis of the Association of Official Analytical Chemists.

[17] Belote B.L., Soares I., Tujimoto-Silva A., Sanches A.W., Kraieski A.L., Santin E. (2019). Applying I see inside histological methodology to evaluate gut health in broilers challenged with *Eimeria*. Vet. Parasitol..

[18] Kraieski A.L., Hayashi R.M., Sanches A., Almeida G.C., Santin E. (2017). Effect of aflatoxin experimental ingestion and *Eimeira* vaccine challenges on intestinal histopathology and immune cellular dynamic of broilers: Applying an Intestinal Health Index. Poult. Sci..

[19] Martin P., Bateson P. (2007). Measuring Behaviour.

[20] Bligh E.G., Dyer W.J. (1959). A rapid method of total lipid extraction and purification. Can. J. Biochem. Physiol..

[21] Hartman L., Lago R.C. (1973). Rapid preparation of fatty acid methyl esters from lipids. Lab. Pract..

[22] Simionato J.I., Garcia J.C., dos Santos G.T., Oliveira C.C., Visentainer J.V., de Souza N.E. (2015). Additions and corrections—Validation of the determination of fatty acids in milk by gas chromatography. J. Braz. Chem. Soc..

[23] Pekas J.C. (1968). Versatile swine laboratory apparatus for physiologic and metabolic studies. J. Anim. Sci..

[24] Adeola O., Lewis A.J., Southern L.L. (2000). Digestion and balance techniques in pigs. Swine Nutrition.

[25] Sakomura N.K., Rostagno H.S. (2016). Métodos de Pesquisa em Nutrição de Monogástricos.

[26] Norkeaw R., Arjin C., Sartsook A., Chaiwang N., Mekchay S., Sommano S.R., Ruksiriwanich W., Sringarm K. (2021). Antioxidant activities of plant extracts and essential oil-cyclodextrin complexes and their effect on lipid accumulation in porcine adipocytes in vitro. Vet. Integr. Sci..

[27] Orengo J., Hernández F., Martínez-Miró S., Sánchez C.J., Rubio C.P., Madrid J. (2021). Effects of commercial antioxidants in feed on growth performance and oxidative stress status of weaned piglets. Animals.

[28] Eliopoulos C., Papadomichelakis G., Voitova A., Chorianopoulos N., Haroutounian S.A., Markou G., Arapoglou D. (2024). Improved Antioxidant Blood Parameters in Piglets Fed Diets Containing Solid-State Fermented Mixture of Olive Mill Stone Waste and *Lathyrus clymenum* Husks. Antioxidants.

[29] Liu A., Li Z., Jin X., Wu Q., Hu H., Zhang C. (2022). An encapsulated organic acid and essential oil mixture improves the intestinal health of weaned piglets by altering intestinal inflammation and antioxidative capacity. Animals.

[30] Bilal R.M., Liu C., Zhao H., Wang Y., Farag M.R., Alagawany M., Hassan F.-U., Elnesr S.S., Elwan H.A.M., Qiu H. (2021). Olive oil: Nutritional applications, beneficial health aspects and its prospective application in poultry production. Front. Pharmacol..

[31] Zou X., Zeng M., Zheng Y., Zheng A., Cui L., Cao W., Wang X., Liu J., Xu J., Feng Z. (2023). Comparative study of hydroxytyrosol acetate and hydroxytyrosol in activating phase II enzymes. Antioxidants.

[32] Klem T.B., Bleken E., Morberg H., Thoresen S.I., Framstad T. (2010). Hematologic and biochemical reference intervals for Norwegian crossbreed grower pigs. Vet. Clin. Pathol..

[33] Elmaksoud H.A.A., Motawea M.H., Desoky A.A., Elharrif M.G., Ibrahimi A. (2021). oxidative stress and apoptosis resulted in ulcerative colitis. Biomed. Pharmacother..

[34] Zhang L., Long S., Wang H., Piao X. (2023). Dietary 25-hydroxycholecalciferol modulates gut microbiota and improves the growth. Front. Microbiol..

[35] Ferlisi F., De Ciucis C.G., Trabalza-Marinucci M., Fruscione F., Mecocci S., Franzoni G., Zinellu S., Galarini R., Razzuoli E., Cappelli K. (2024). Olive mill waste-water extract enriched in hydroxytyrosol and tyrosol modulates host–pathogen interaction in IPEC-J2 cells. Animals.

[36] Gómez G., Laviano H.D., García-Casco J.M., Escudero R., Muñoz M., Heras-Molina A., González-Bulnes A., Óvilo C., López-Bote C., Rey A.I. (2023). Different effect of vitamin E or hydroxytyrosol supplementation to sow’s diet on oxidative status and performances of weaned piglets. Antioxidants.

[37] Lu Y., Zhao M., Mo J., Lan G., Liang J. (2022). Dietary supplementation ellagic acid on the growth. Front. Vet. Sci..

[38] Rodríguez-Morató J., Xicota L., Fitó M., Farré M., Dierssen M., De La Torre R. (2015). Potential Role of Olive Oil Phenolic Compounds in the Prevention of Neurodegenerative Diseases. Molecules.

[39] Silva S., Sepodes B., Rocha J., Direito R., Fernandes A., Brites D., Freitas M., Fernandes E., Bronze M., Figueira M. (2015). Protective effects of hydroxytyrosol-supplemented refined olive oil in animal models of acute inflammation and rheumatoid arthritis. J. Nutr. Biochem..

[40] Piotti P., Pierantoni L., Albertini M., Pirrone F. (2024). Veterinary Clinics of North America: Small Animal Practice. Vet. Clin. N. Am. Small Anim. Pr..

[41] Nordgreen J., Edwards S.A., Boyle L.A., Bolhuis J.E., Veit C., Sayyari A., Marin D.E., Dimitrov I., Janczak A.M., Valros A. (2020). A proposed role for pro-inflammatory cytokines in damaging behavior in pigs. Front. Vet. Sci..

[42] Huang Y., He Z., Li H., Li F., Wu Z. (2012). Effect of antioxidant on the fatty acid composition and lipid oxidation of intramuscular lipid in pressurized pork. Meat Sci..

[43] Su H., Shi P., Shen Z., Meng H., Meng Z., Han X., Chen Y., Fan W., Fa Y., Yang C. (2023). High-level production of nervonic acid in the oleaginous yeast *Yarrowia lipolytica* by systematic metabolic engineering. Commun. Biol..

[44] Fan Y., Meng H.-M., Hu G.-R., Li F.-L. (2018). Biosynthesis of nervonic acid and perspectives for its production by microalgae and other microorganisms. Appl. Microbiol. Biotechnol..

[45] Li Q., Chen J., Yu X., Gao J.-M. (2019). A mini review of nervonic acid: Source. Food Chem..

[46] Namiecinska M., Piatek P., Lewkowicz P. (2024). Nervonic acid synthesis substrates as essential components in profiled lipid supplementation for more effective central nervous system regeneration. Int. J. Mol. Sci..

[47] Rossi R., Pastorelli G., Cannata S., Tavaniello S., Maiorano G., Corino C. (2013). Effect of long term dietary supplementation with plant extract on carcass characteristics meat quality and oxidative stability in pork. Meat Sci..

[48] Paiva-Martins F., Barbosa S., Pinheiro V., Mourão J.L., Outor-Monteiro D. (2009). The effect of olive leaves supplementation on the feed digestibility. Meat Sci..

[49] Ferlisi F., Tang J., Cappelli K., Trabalza-Marinucci M. (2023). Dietary supplementation with olive oil co-products rich in polyphenols: A novel nutraceutical approach in monogastric animal nutrition. Front. Vet. Sci..

