# Supplementary material for: Effects of Adding Hydroxytyrosol to the Diet of Pigs in the Nursery Phase on Growth Performance, Biochemical Markers, and Fatty Acid Profile
Source: Animals (Basel). 2025 Aug 1;15(15):2268. doi: 10.3390/ani15152268 (PMC12345483; doi:10.3390/ani15152268)
Supplement: Supplementary file 1 [file animals-15-02268-s001.zip › animals-3787917-supplementary.pdf]

Supplementary Materials

Dry -bulb temperature (DBT) and relative humidity (RH)

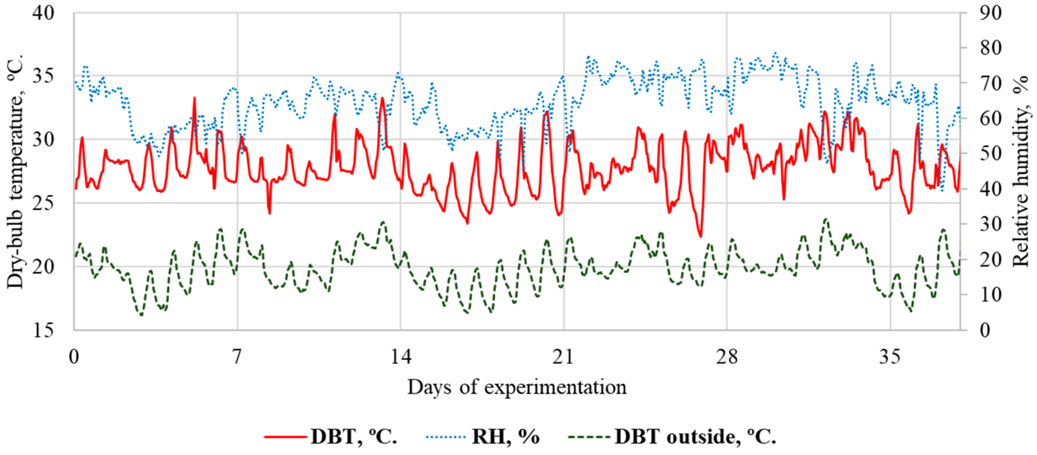

Source: Author, 2024.

Figure S1. Environmental variables recorded during the experimental period.
